# Hybrid cells derived from breast epithelial cell/breast cancer cell fusion events show a differential RAF-AKT crosstalk

**DOI:** 10.1186/1478-811X-10-10

**Published:** 2012-04-09

**Authors:** Cem Özel, Jeanette Seidel, Sönke Meyer-Staeckling, Burkhard H Brandt, Bernd Niggemann, Kurt S Zänker, Thomas Dittmar

**Affiliations:** 1 Zentrum für Biomedizinische Ausbildung und Forschung der UWH (ZBAF), Institute of Immunology, Witten/Herdecke University, Stockumer Str. 10, 58448 Witten, Germany; 2 Institute of Tumorbiology, University Hospital Hamburg Eppendorf, Martinistr. 52, 20246 Hamburg, Germany; 3 Institute of Clinical Chemistry, University Hospital Schleswig Holstein, Michaelisstr. 5, 24105 Kiel, Germany

## Abstract

**Background:**

The biological phenomenon of cell fusion has been linked to several characteristics of tumour progression, including an enhanced metastatogenic capacity and an enhanced drug resistance of hybrid cells. We demonstrated recently that M13SV1-EGFP-Neo breast epithelial cells exhibiting stem cell characteristics spontaneously fused with MDA-MB-435-Hyg breast cancer cells, thereby giving rise to stable M13MDA435 hybrid cells, which are characterised by a unique gene expression profile and migratory behaviour. Here we investigated the involvement of the PLC-β/γ1, PI3K/AKT and RAS-RAF-ERK signal transduction cascades in the EGF and SDF-1α induced migration of two M13MDA435 hybrid cell clones in comparison to their parental cells.

**Results:**

Analysis of the migratory behaviour by using the three-dimensional collagen matrix migration assay showed that M13SV1-EGFP-Neo cells as well as M13MDA435 hybrid cells, but not the breast cancer cell line, responded to EGF stimulation with an increased locomotory activity. By contrast, SDF-1α solely stimulated the migration of M13SV1-EGFP-Neo cells, whereas the migratory activity of the other cell lines was blocked. Analysis of signal transduction cascades revealed a putative differential RAF-AKT crosstalk in M13MDA435-1 and -3 hybrid cell clones. The PI3K inhibitor Ly294002 effectively blocked the EGF induced migration of M13MDA435-3 hybrid cells, whereas the EGF induced locomotion of M13MDA435-1 hybrid cells was markedly increased. Analysis of RAF-1 S259 phosphorylation, being a major mediator of the negative regulation of RAF-1 by AKT, showed decreased pRAF-1 S259 levels in LY294002 treated M13MDA435-1 hybrid cells. By contrast, pRAF-1 S259 levels remained unaltered in the other cell lines. Inhibition of PI3K/AKT signalling by Ly294002 relieves the AKT mediated phosphorylation of RAF-1, thereby restoring MAPK signalling.

**Conclusions:**

Here we show that hybrid cells could evolve exhibiting a differential active RAF-AKT crosstalk. Because PI3K/AKT signalling has been chosen as a target for anti-cancer therapies our data might point to a possible severe side effect of AKT targeted cancer therapies. Inhibition of PI3K/AKT signalling in RAF-AKT crosstalk positive cancer (hybrid) cells could result in a progression of these cells. Thus, not only the receptor (activation) status, but also the activation of signal transduction molecules should be analysed thoroughly prior to therapy.

## Background

The biological phenomenon of cell fusion plays a fundamental role in a plethora of physiological events as well as pathophysiological events (an overview is given in [1]). In cancer, the fusion between tumour cells and tumour cells as well as tumour cells and normal cells, has been linked to several characteristics of tumour progression, including an enhanced metastatogenic capacity and an enhanced drug resistance [2-8]. Additionally, cell fusion has also been suggested as one process how cancer stem cells could originate [9-12].

The hypothesis that cell fusion might play a crucial role in tumour progression was postulated by the German Physician Otto Aichel about 100 years ago [4]. In his outstanding work, Aichel proposed that fusion between tumour cells and leukocytes could give rise to tumour cells exhibiting leukocyte characteristics, such as the ability to migrate [4]. The subject "cell fusion in cancer" is still controversially debated [10,11,13,14] and till now considerably less is known how the multi-step process of cell fusion [15] between tumour cells and other cells is regulated, which also belongs to the molecules being involved in this process. Most of the identified fusion-mediating molecules, e.g., CD47 [16], CD44 [16], CD200 [17], and syncytin-1 [18-20], are expressed on cell types, such as macrophages, knowing to undergo cell fusion during physiological processes. Recent data indicate that fusion events were increased about 10 to 100-fold in liver, brain and intestine in chronic inflammatory conditions [21-23] suggesting that inflammation might be a strong trigger for cell fusion. Since tumour tissue resembles chronically inflamed tissue [24-26] the tumour microenvironment itself might thus provide a surrounding area that trigger the fusion of tumour cells with other cells.

Recent data of the past years provided evidence that cell fusion is a common event in cancer [27-29]. By using a parabiosis model, a GFP mouse was surgically joined with an APC^Min/+ ^mouse, Powell et al. demonstrated recently that cell fusion in tumour tissue between cancer cells and macrophages and B- and T-Lymphocytes occurred in vivo [28]. Analysis of the gene expression profile of hybrid cells showed that these cells retain a transcriptome identity characteristic of both parental derivatives, while also expressing a unique subset of transcripts, which may have important consequences for tumorigenesis and metastogenesis [28]. A unique gene expression pattern was further described for hybrid cells derived from weakly malignant Cloudman S91 melanoma cells and macrophages [30,31], human breast epithelial cells exhibiting stem cell characteristics and breast cancer cells [7], and murine 67NR mammary carcinoma cells and mouse bone marrow-derived cells (BMDCs) [8]. Rizvi and colleagues provided evidence that murine BMDCs restore murine intestinal tissue in a long term repopulation fashion suggesting that BMDCs have fused with intestinal stem and/or progenitor cells [29]. These data let assume that macrophages and/or BMDCs will preferentially fuse with cancer stem/progenitor cells in order to ensure tumour tissue homeostasis.

Recently, we demonstrated that human mammary epithelial cells exhibiting stem cell characteristics spontaneous fuse with human breast cancer cells, thereby giving rise to stable hybrids [32]. These cells were characterised by a nearly doubled chromosomal number and an increased proliferation rate [10] as well as a unique gene expression profile concomitant with an altered migratory behaviour [7]. In the present work we analysed the role of three major cell migration related signal transduction cascades - PLC-γ1, PI3K/AKT, RAS-RAF-MAPK - in two hybrid cell lines and their parental cells. Since these hybrid cell lines were also positive for the stromal cell-derived factor-1α (SDF-1α) receptor CXCR4 we additionally investigated both the effect of EGF and SDF-1α. Our data show that each hybrid cell line exhibited a unique signal transduction cascade kinetics pattern including a differential activity of the RAF-AKT crosstalk.

## Results

### Flow cytometry analysis of EGFR family members and CXCR4

**Figure 1:**
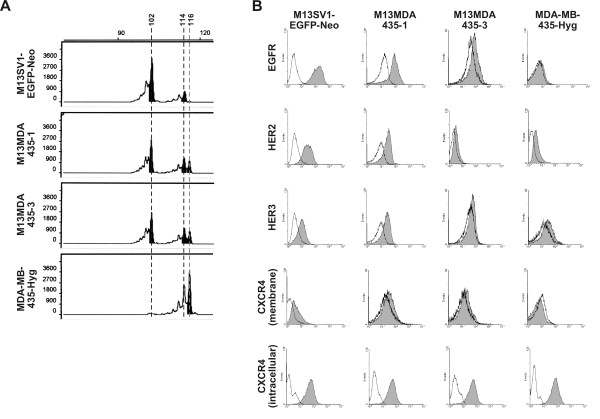
**STR-analysis and flow cytometry**. A) Shown are representative data for the D16S402 PCR. Breast epithelial cell specific alleles are marked by a black dashed line, whereas a grey dashed line marks breast cancer cell specific alleles. Black filled histograms represent the predominant detected alleles. An overlay of parental alleles is clearly detectable in hybrid cells. B) Flow cytometry analysis of EGFR, HER2, HER3, and CXCR4 (membrane bound and intracellular). Shown are representative data of one of at least three independent measurements. White histogram: isotype control, grey histogram: specific antibody.

In the present study we investigated the migratory activity of M13MDA435-1 and -3 hybrid clones and their parental cells (M13SV1-EGFP-Neo breast epithelial cells and MDA-MB-435-Hyg breast cancer cells) in response to EGF and SDF-1α. Both hybrid clones derived from spontaneous fusion events between M13SV1-EGFP-Neo cells and MDA-MB-435-Hyg cells and were isolated by a dual antibiotic selection procedure [7]. Parental cells were co-cultured for 24 h prior to addition of antibiotics [7]. The origin of hybrid cells by cell fusion and not horizontal gene transfer was proven by short tandem repeat (STR) analysis, whereby specific sequences on different chromosomes were analyzed. STR analysis revealed an overlap of parental alleles in hybrid clone M13MDA435-3 (Figure 1A; Table 1), which is in accordance to M13MDA435-1 hybrids [7]. EGFR, HER2, HER3 and membrane bound CXCR4 expression levels of M13SV1-EGFP-Neo breast epithelial cells, MDA-MB-435-Hyg breast cancer cells and M13MDA435-1 hybrid cells were comparable to previously published data [7]. Compared to hybrid clone M13MDA435-1 MDA-MB-435-3 hybrid cells showed weaker expression levels of EGFR, HER2 and HER3, but comparable levels of membrane bound CXCR4 (Figure 1B). Because CXCR4 could also be stored in intracellular vesicles [33], we additionally performed flow cytometry studies to look for intracellular localized CXCR4 in hybrid cells and parental cells. Data are summarized in Figure 1B and clearly show that all cells harbour similar levels of intracellular CXCR4, which is in accordance to PCR data (data not shown).

**Table 1:** Genotypic analysis of M13SV1-EGFP-Neo/MDA-MB-435-Hyg hybrid cells

Primer	M13SV1-EGFP-Neo	M13MDA-435-1	M13MDA-435-3	MDA-MB-435-Hyg
D16S402	102	102	102	
	
	114	114	114	
	
		116	116	116

NEFL^a^ D17S855^a^ D13S153^a^	94	94	94	94
	
	143	143	143	
	
	149	149	149	149
	
		173	173	173
	
	175	175	175	

CASSR1	114	114	114	114
	
	132			

^a ^These primer pairs were used for the multiplex PCR

### Migration analysis of breast epithelial cells/breast cancer cell hybrids

**Figure 2:**
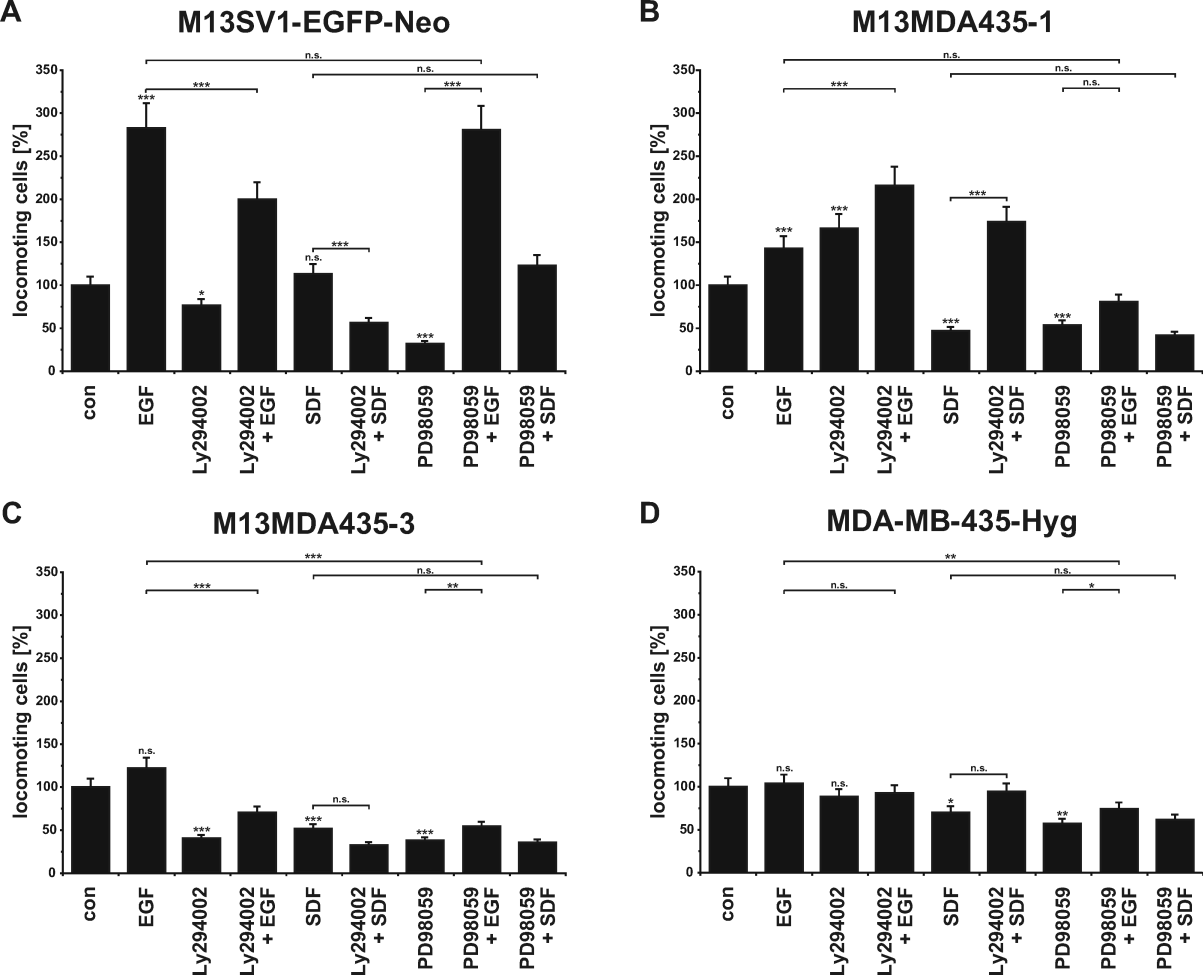
**Cell migration data**. The migratory activity was analyzed using the 3D collagen matrix migration assay combined with time-lapse video-microscopy. For a better comparison of the EGF and SDF-1α specific, values of each cell type were calculated in relation to untreated control values, which were set to 100%. Cells were stimulated with 100 ng/ml EGF; 1 μg/ml SDF-1α, 500 nM L294002, and 500 nM PD98059. A) M13SV1-EGFP-Neo breast epithelial cells, B) M13MDA435-1 hybrid cells, C) M13MDA435-3 hybrid cells, D) MDA-MB-435-Hyg breast cancer cells. Statistical significance was calculated using Student's *t*-test: n.s. = not significant; * = *p *< 0.05; ** = *p *< 0.01; *** = *p *= 0.001.

Due to expression of EGFR family members and CXCR4 the migratory activity of M13MDA435 hybrid cells in dependence of EGF and SDF-1α stimulation was investigated by using the three-dimensional collagen matrix migration assay [7,34-36]. M13SV1-EGFP-Neo breast epithelial cells exhibiting stem cell properties responded well to EGF treatment with an increased locomotory activity, which is in accordance to previously published data [7] (Figure 2A). Likewise, MDA-MB-435-Hyg breast cancer cells did not respond to EGF stimulation (Figure 2D). Both hybrid cell lines showed an increased migratory activity upon EGF stimulation (Figure 2B, C). Compared to M13MDA435-3 hybrid cells the EGF induced locomotory activity of M13MDA435-1 cells was slightly higher (Figure 2B, C), which might be attributed to the cells higher EGFR, HER2, and HER3 expression levels (Figure 1B).

M13SV1-EGFP-Neo breast epithelial cells exhibiting stem cell characteristics responded to SDF-1α stimulation with a slightly, but not significantly increased locomotory activity (Figure 2A). Interestingly, the migratory activity of MDA-MB-435-Hyg breast cancer cells was decreased by SDF-1α (Figure 2D). Because cells possess marked levels of intracellular CXCR4, but not membrane bound CXCR4, we assume that upon SDF-1α stimulation CXCR4 from intracellular compartments is transported to the surface. In accordance to MDA-MB-435-Hyg breast cancer cells the migratory activity of both hybrid cell lines was effectively blocked by SDF-1α (Figure 2B, C).

### Calcium measurements

**Figure 3:**
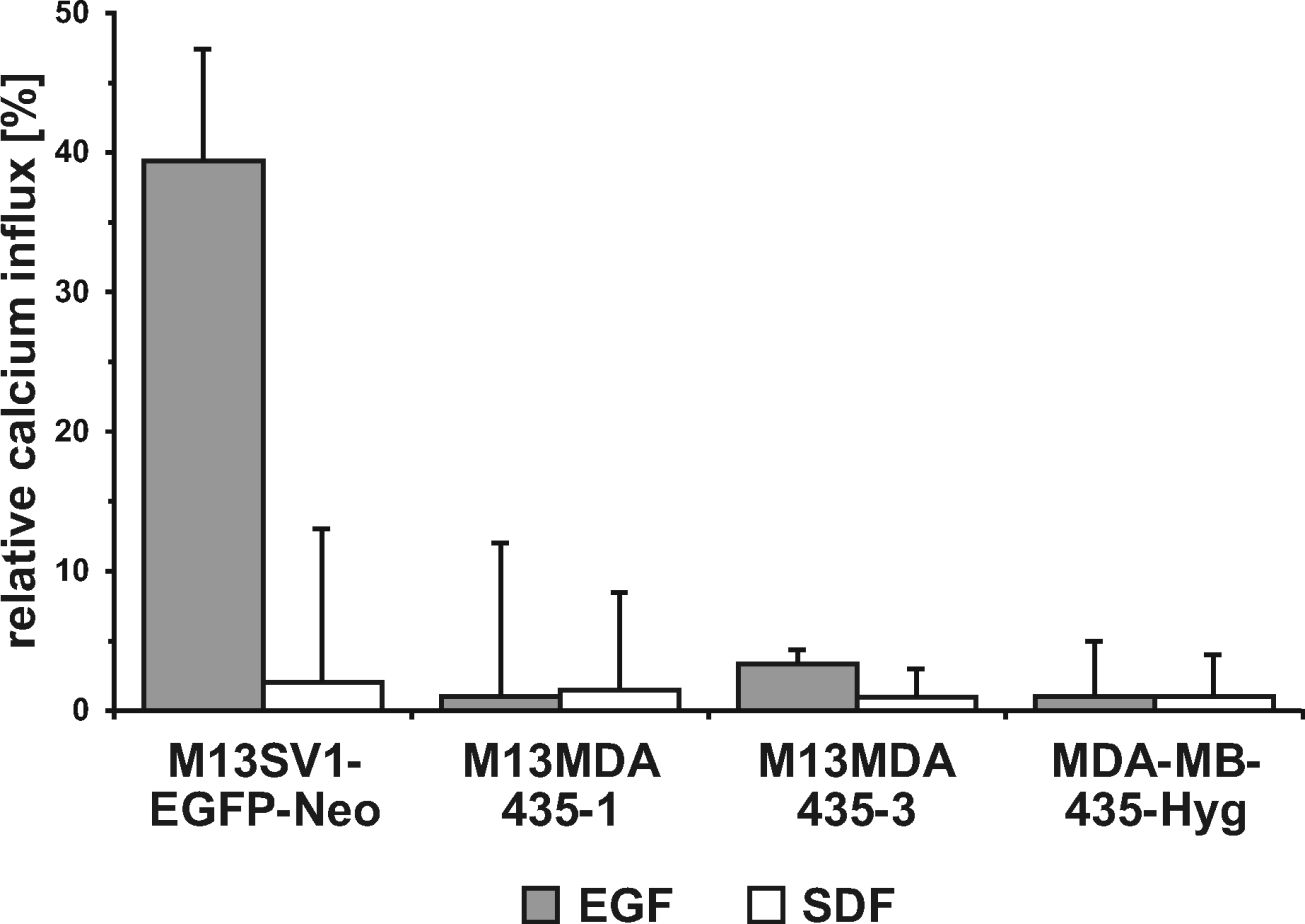
**Calcium measurements**. The diagram shows the mean calcium influx in EGF and SDF-1α treated cells. An increase in cytosolic calcium concentrations was only observed in EGF stimulated M13Sv1-EGFP-Neo cells. Shown are the mean of at least three independent experiments.

In order to investigate whether the differential susceptibility of all analysed cells towards EGF and SDF-1α was attributed to a differential engagement of signal transduction pathways we first conducted flow cytometry-based calcium measurements to determine whether PLC-β/γ1 signalling was activated upon EGF and SDF-1α stimulation. Data are summarized in Figure 3 and clearly show that only EGF stimulation of M13SV1-EGFP-Neo cells resulted in increased intracellular calcium concentrations.

### M13MDA435 hybrids exhibit a differential PI3K/AKT and MAPK^p42/44 ^signalling

**Figure 4:**
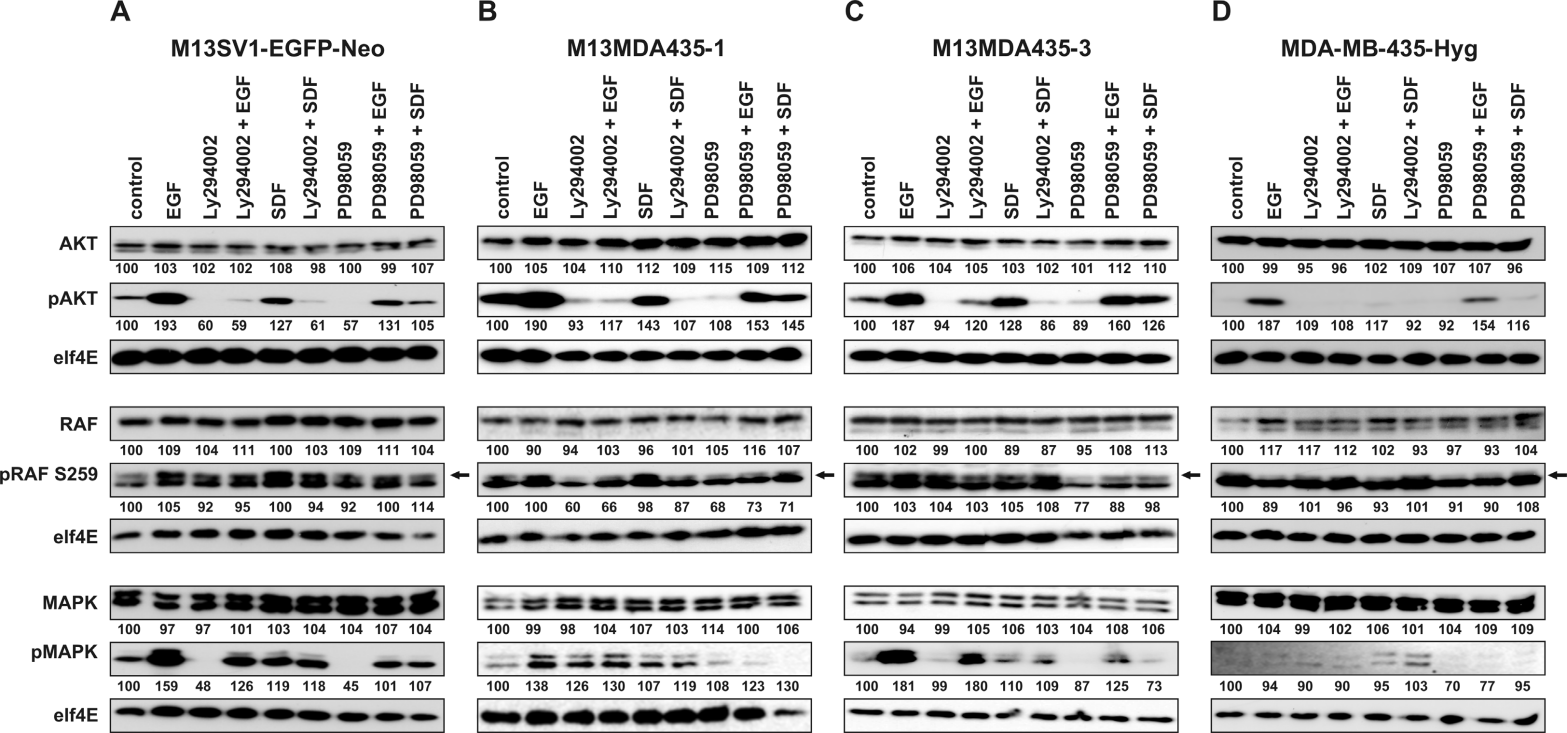
**Western Blot analysis of PI3K/AKT and RAS-RAF-MAPK signalling**. The effect of EGF and SDF-1α on induction of PI3K/AKT and RAS-RAF-MAPK signalling was analysed by Western Blot. Cells were stimulated for 5 minutes with 100 ng/ml EGF and 1 μg/ml SDF-1α. In case of inhibitor treatment cells were preincubated for 30 minutes with 500 nM Ly294002 and 500 nM PD98059. The downstream effect of EGF and SDF-1α stimulation on the AKT and MAP-kinase pathways was determined by phosphorylation of AKT and MAPK^p42/44^. To investigate for the putative RAF-AKT crosstalk RAF-1 expression and RAF-1 phosphorylation at position S259 was determined. The arrow marks pRAF-1 S259. elf4E served as loading control. Shown are representative Western-Blot data of three independent experiments. A) M13SV1-EGFP-Neo breast epithelial cells, B) M13MDA435-1 hybrid cells, C) M13MDA435-3 hybrid cells, D) MDA-MB-435-Hyg breast cancer cells. Bands were densitometric analysed by using the ImageJ software http://rsbweb.nih.gov/ij/. Relative intensities of protein and phosphoprotein expression were calculated in relation to elf4E and controls, which were set to 100%.

We next analysed the function of PI3K/AKT and MAPK^p42/44 ^signalling by using the well characterised pharmacological inhibitors Ly294002 (PI3K inhibitor) and PD98059 (MAPK^p42/44 ^inhibitor). Ly294002 treatment inhibited both the EGF and SDF-1α induced migration of M13SV1-EGFP-Neo breast epithelial cells exhibiting stem cells characteristics (Figure 2A). In contrast to this, PD98059 solely blocked the spontaneous migration of M13SV1-EGFP-Neo cells, but had no inhibitory effect on the EGF and SDF-1α induced migration (Figure 2A). Stimulation of M13SV1-EGFP-Neo cells with EGF resulted in increased pAKT and pMAPK^p42/44 ^levels, which were markedly blocked by Ly294002 and PD98059 treatment (Figure 4A). Similar data were obtained for SDF-1α, whereby the increase in AKT and MAPK^p42/44 ^phosphorylation was rather moderate as compared to EGF stimulated cells (Figure 4A). Likewise, SDF-1α mediated phosphorylation of AKT and MAPK^p42/44 ^was effectively blocked by both Ly294002 and PD98059 treatment (Figure 4A).

Treatment of MDA-MB-435-Hyg breast cancer cells with Ly294002 and PD98059 showed that only PD98059 significantly impaired the cells' spontaneous migration (Figure 2D). Ly294002 blocked the inhibitory effect of SDF-1α on MDA-MB-435-Hyg cells, whereby this effect was not significant (Figure 2D). Western Blot analysis of EGF and stimulated MDA-MB-435-Hyg cells revealed increased pAKT levels upon EGF stimulation, which were effectively blocked by Ly294002 (Figure 4D). By contrast, no MAPK^p42/44 ^phosphorylation was detected in EGF treated MDA-MB-435-Hyg cells (Figure 4D). Stimulation of MDA-MB-435-Hyg breast cancer cells with SDF-1α did neither result in AKT nor in MAPK^p42/44 ^phosphorylation (Figure 4D).

Analysis of M13MDA435-1 and M13MDA435-3 hybrid cells resulted in totally different outcomes. The spontaneous as well as the EGF induced migration of M13MDA435-3 hybrid cells were markedly impaired by both Ly294002 and PD98059 (Figure 2C). Likewise, the migratory activity of MDA-MB-435-Hyg breast cancer cells co-treated with either SDF-1α and Ly294002 or SDF-1α and PD98059 was comparable to the locomotory behaviour of cells treated with the appropriate inhibitor alone (Figure 2C). These data indicate that both inhibitors rather blocked the spontaneous migration of M13MDA435-3 hybrid cells than the factor-specific mediated migratory activities. Otherwise, one would have expected a more profound inhibitory effect of Ly294002 on the EGF induced migration. Western Blot data of M13MDA435-3 hybrid cells were comparable to M13SV1-EGFP-Neo cells. Both EGF and SDF-1α treatment resulted in increased pAKT and pMAPK^p42/44 ^levels, which were blocked by Ly294002 and PD98059 (Figure 4C).

However, in contrast to M13MDA435-3 hybrid cells, the PI3K inhibitor Ly294002 potently induced the spontaneous migration as well as the EGF induced migration of M13MDA435-1 hybrid cells (Figure 2B). Moreover, Ly294002 effectively reverted the inhibitory effect of SDF-1α on the migration of this hybrid clone (Figure 2B) clearly indicating the stimulatory effect of this inhibitor on the migratory activity of M13MDA435-1 hybrid cells. PD98059 inhibited both the spontaneous and the EGF induced migration (Figure 2B), which is in accordance to M13MDA435-3 hybrid cells, but opposite to M13SV1-EGFP-Neo breast epithelial cells (Figure 2A,C). The locomotory activity of PD98059 treated M13MDA435-1 hybrid cells was comparable to the migratory activity of solely SDF-1α treated as well as SDF-1α and PD98059 co-treated cells (Figure 2B). In accordance to all other analysed cells EGF and SDF-1α stimulation resulted in increased pAKT levels in M13MDA435-1 hybrid cells, which were effectively blocked by Ly294002 (Figure 4B). Likewise, both EGF and SDF-1α stimulation resulted in increased pMAPK^p42/44 ^levels in M13MDA435-1 hybrid cells, which were impaired by PD98059 (Figure 4B). Interestingly, in contrast to M13SV1-EGFP-Neo breast epithelial cells and M13MDA435-3 hybrids phosphorylated MAPK^p42/44 ^levels were also clearly detectable in Ly294002 treated M13MDA435-1 hybrid cells (Figure 4B), which might be an explanation for the stimulatory effect of this inhibitor on the migratory activity of these cells.

### Do M13MDA435-1 hybrid cells possess an altered RAF-AKT crosstalk?

The differential effect of the PI3K inhibitor Ly294002 on the EGF-induced migratory activity of M13DA435-1 and M13MDA435-3 hybrid cells might be attributed to a differential active RAF-AKT crosstalk [37,38]. AKT mediated phosphorylation of RAF-1 at position S259 is consistent with RAF-1 inactivation [38]. Thus Western Blot studies were conducted to determine pRAF-1 S259 levels in cells. In M13SV1-EGFP-Neo breast epithelial cells similar amounts of pRAF-1 S259 were detected irrespective of applied conditions (Figure 4A), which is opposite to MDA-MB-435-Hyg breast cancer cells. Here, no or solely very faint pRAF-1 S259 levels were detected (Figure 4D). Analysis of M13MDA435-1 and M13MDA435-3 hybrid cells revealed different pRAF-1 S259 levels. In accordance to M13SV1-EGFP-Neo cells, similar amounts of pRAF-1 S259 were detected in M13MDA435-3 hybrid cells irrespective of the applied conditions (Figure 4C). By contrast, decreased pRAF-1 S259 levels were detected in M13MDA435-1 hybrid cells treated with Ly294002 alone or in combination with EGF or SDF-1α (Figure 4B). These data likely support the putative RAF-AKT crosstalk in M13MDA435-1 hybrid cells. Inhibition of AKT signalling by Ly294002 impairs AKT mediated phosphorylation of RAF-1 at position S259, thereby activating RAF-MAPK signalling.

Interestingly, we noticed slightly decreased pRAF-1 S259 levels in PD98059 treated hybrid cell lines (Figure 4). This effect was reproducible and might be attributed to an MAPKp42/44 RAF-1 negative feedback loop [39,40]. Due to inhibition of MAPK^p42/44 ^signalling by PD98059 this negative feedback is abrogated thus preventing RAF-1 S259 phosphorylation.

## Discussion

In the present study we investigated the migratory activity of the two hybrid cell lines M13MDA435-1 and M13MDA435-3 in dependence of EGF and SDF-1α stimulation in comparison to M13SV1-EGFP-Neo breast epithelial cells exhibiting stem cell characteristics and HS578T-Hyg breast cancer cells. Our data show that each hybrid clone exhibited a unique EGF and SDF-1α mediated migratory activity, which was most likely attributed to a unique signal transduction cascade kinetics pattern in each hybrid cell clone.

Cell migration is a complex process and is directed by the interplay of several signal transduction pathways initiated by various ligands such as growth factors, chemokines and extracellular matrix components that activate growth factor receptors, chemokine receptors and integrins [41,42]. The involvement of different pro-migratory pathways initiated by different inducers is nicely seen in M13SV1-EGFP-Neo breast epithelial cells exhibiting stem cell properties. EGF treatment resulted in a markedly induced migratory activity of the cells. In accordance to M13MDA435-3 hybrid cells the PI3K inhibitor Ly294002 impaired both the spontaneous and EGF induced migration of M13SV1-EGFP-Neo cells. However, the inhibitory effect of Ly294002 on the spontaneous migration of the cells was rather moderate (about 25%), whereas the inhibitory effect of Ly294002 on the EGF induced migration of M13SV1-EGFP-Neo cells was much higher (about 90%). This indicates that inhibition of PI3K/AKT signalling impairs both the spontaneous and the EGF induced migration.

Since only M13SV1-EGFP-Neo breast epithelial cells showed an EGF-dependent calcium influx and EGFR/HER2/PLC-γ1 signalling has been suggested as a key regulatory step in cell migration [34,43] we conclude that the markedly increased migratory activity of the cells in response to EGF was attributed to the induction of this signal transduction cascade mediated by EGFR/HER2 heterodimer signalling. Moreover, data of Falasca and colleagues provided evidence that PLC-γ1 signalling does also depend on PI3K activity [44]. PI3K generates phosphatidylinositol-3,4,5-triphosphate to which PLC-γ1 binds with its pleckstrin homology domain [44]. By doing so PLC-γ1 translocates to the plasma membrane and is subsequently activated by tyrosine phosphorylation [44]. PI3K is highly activated by HER2/HER3 signalling [45,46]. In the context of breast cancer HER2/HER3 heterodimer signalling has been referred to function as an oncogenic unit [45]. Thus inhibition of PI3K activity by Ly294002 also impairs PLC-γ1 signalling, which might be a suitable explanation for the effective inhibition of Ly294002 on the EGF induced migration of M13SV1-EGFP-Neo cells initiated by the interplay of EGFR/HER2/PLC-γ1 and HER2/HER3/PI3K signalling. Similar findings were recently demonstrated by Balz et al. demonstrating that the EGF induced calcium influx concomitant with the migratory activity was markedly decreased in EGFR/HER2/HER3 positive MDA-HER2 breast cancer cells treated with the PI3K inhibitor wortmannin [47].

This assumption is further substantiated by PD98059 data. MAPK signalling does not interfere with PLC-γ1 signalling and because of that inhibition of MAPK signalling did not result in a decreased EGF-induced migration of M13SV1-EGFP-Neo cells. Moreover, these data further show the differential roles of signal transduction cascades in regulating cell migration. Even though MAPK signalling is involved in the spontaneous migration of M13SV1-EGFP-Neo cells, the inhibition of this pathway by PD98059 is superimposed by the PLC-γ1 signalling pathway.

Of interest was the finding of the diametrically opposed effect of the PI3K inhibitor Ly294002 on M13MDA435-1 and M13MDA435-3 hybrid cells. While Ly294002 effectively inhibited the migratory activity of M13MDA435-3 hybrid cells, the migration of M13MDA435-1 hybrids was potently stimulated by this compound. We assume that this effect might be attributed to a differential RAF-AKT crosstalk [37] in both hybrid cell lines. The RAF-AKT crosstalk describes the interplay between AKT and RAF-1 signalling, whereby a strong PI3K/AKT activation leads to a AKT dependent inhibition of RAF-1 concomitant with abrogation of MAPK^p42/44 ^signalling [37]. Inhibition of AKT signalling, e.g., by Ly294002 treatment, relieves this block and restores RAF-1-MAPK^p42/44 ^signalling [37]. Moelling et al. observed increased phosphorylated MAPK^p42/44 ^levels in IGF and Ly294002 co-treated cells [37], which is similar to our work.

The putative RAF-AKT crosstalk in M13MDA435-1 hybrid cells was further validated by Western Blot analysing RAF-1 phosphorylation at position S259, which is consistent with AKT mediated RAF-1 inactivation [38]. In fact, Western Blot analysis of LY294002 treated M13MDA435-1 hybrid cells showed decreased pRAF-1 S259 levels, which were neither detected in M13MDA435-3 hybrid cells nor in the parental cells. This finding would be in view with the assumption of an active RAF-AKT crosstalk in M13MDA435-1 hybrid cells. Treatment of M13MDA435-1 cells with Ly294002 blocks PI3K/AKT signalling, thereby impairing AKT mediated RAF-1 S259 phosphorylation, which in turn restores RAF-1-MAPK^p42/44 ^signalling including MAPK^p42/44 ^phosphorylation.

Whether the differential activity of the RAF-AKT crosstalk was attributed to the cell fusion process or to another mechanism is unclear. Cell fusion is a random process associated with chromosomal instability, loss of single chromosomes and aneuploidy in emerging hybrid cells [6,10,11]. As a consequence each hybrid clone originate individually and because of that each hybrid clone exhibit a unique gene expression profile concomitant with a unique signal transduction cascade pattern. On the other hand, Rommel and colleagues showed that the regulation of RAF-AKT crosstalk also depended on the differentiation state of the cell [48]. AKT activation inhibited the RAF-MAPK pathways in differentiated myotubes, but not in their myoblast precursors, which might be attributed to a stage-specific ability of AKT to form a complex with RAF [48]. Thus, the deactivated RAF-AKT crosstalk in M13MDA435-3 hybrid cells might be a relict of stem cell-like phenotype of M13SV1-EGFP-Neo cells. In this cell line no RAF-AKT crosstalk was observed. Activation of the RAF-AKT crosstalk in M13MDA435-1 hybrid cells might thus be attributed to some kind of a maturation/differentiation process that may have occurred in this hybrid cell line during its evolution.

The finding that both hybrid cells responded to EGF with an increased migratory activity, whereas the parental tumour cell line MDA-MB-435-Hyg did not, indicate the potency of cell fusion in changing the fate of cancer cells. Moreover, cell fusion could not only revert the migratory phenotype of a parental cancer cell, but also could give rise to hybrid cells each exhibiting a unique signal transduction cascade pattern. This knowledge would of interest in case of anti-cancer therapies specifically targeting single molecules and pathways. As mentioned above, the HER2/HER3 heterodimer has been referred to act as an oncogenic unit in breast cancer [45,46] due to strong activation of the pro-survival PI3K/AKT pathway. Because of that HER3 has been recommended as a suitable target for novel anti-cancer therapies. For instance, inhibition of HER2/HER3 heterodimer signalling either by shHER3 mediated knock-down or pertuzumab/trastuzumab treatment was correlated with markedly reduced growth of BT474M1 and MDA-MB-175 induced tumours *in vivo *[49]. Similar results were obtained by a Cre-mediated HER3 deletion or by a chemically stabilized HER3 antisense oligonucleotide, in a murine model of mammary carcinoma [50]. While such strategies would effectively block HER3/PI3K/AKT signalling it would be of interest to investigate the effect of this blockage in the hybrid cells used in this study. Inhibition of AKT signalling, e.g., by Ly294002 treatment, can restore RAF-1-MAPK^p42/44 ^signalling [37]. Thus, a similar effect should occur in case of HER3 inhibition, e.g., by using recombinant monoclonal antibodies targeting HER2 and HER3 molecules.

In addition to EGF we also investigated the migratory activity of the cells within the presence of SDF-1α since the parental M13SV1-EGFP-Neo cell line and both hybrid cell lines were slightly positive for CXCR4. The SDF-1α receptor CXCR4 has been linked to the organ-specific metastatic spreading of breast cancer [51,52]. The finding that both hybrid cells were positive for CXCR4 may suggest that cell fusion might be a mechanism how (breast) cancer cells could acquire the ability to metastasise in an organ-specific manner. Several lines of evidence indicated that fusion of tumour cells with other cells, such as macrophages, gave rise to hybrid cells exhibiting an increased metastatogenic capacity [5,6,31,53]. However, cell migration data revealed that the migratory activity of both hybrid cell lines (and the parental breast cancer cell line) was markedly impaired by this chemokine, which is contrary to parental M13SV1-EGFP-Neo breast epithelial cells. Here CXCR4 expression was correlated to a slightly, but not significantly increased migratory activity of the cells upon SDF-1α stimulation.

The SDF-1α receptor CXCR4 belongs to the family of G-protein coupled receptors (GPCRs) [54,55]. Recent data indicated that CXCR4 signalling may not be limited to G_αi _as first thought, but that CXCR4 can couple to other G_α _proteins such as G_αq_, G_αo _and G_αs _[54,56]. Thus, the differential SDF-1α migratory activities of the analysed cells might be attributed to differential expression levels of G_α _subunits. For instance, adenyl cyclase is activated by G_αs_, but blocked by G_αi _[54]. In neutrophil granulocytes the interleukin-8 (IL-8) dependent activation of the adenyl cyclase/PKA pathway has been associated with the induction of a stop-signal, thereby impairing the cells overall and fMLP induced migration in a dose-dependent manner [57]. Activation of the adenyl cyclase/PKA pathway promotes the sequestration of cytosolic calcium in cells [57], thereby impairing cell migration.

Holland and colleagues demonstrated that association of G-protein αβγ-heterotrimers with CXCR4 receptor and induction of a SDF-1α specific signalling did only occur in highly invasive breast cancer cell lines, but not in non-invasive cell lines [58]. Here, the blockade of non-metastatic cell lines seemed to be due to the inability of G protein α and β subunits to form a heterotrimeric complex with CXCR4, which, on the other hand, was observable in highly invasive cell lines [58].

However, both hybrid cell lines as well as the parental MDA-MB-435-Hyg breast cancer cell line responded to SDF-1α treatment with a decreased migratory activity. Thus we assume that these cells harbour a functional CXCR4 receptor coupled with a functional G-protein αβγ-heterotrimer. In addition to heterotrimeric G-protein dependent signals CXCR4, like all GPCRs, is a substrate for G protein receptor kinases (GRKs) [56]. GRK-mediated phosphorylation of CXCR4 creates a binding site for β-arrestins, thereby enabling a heterotrimeric G-protein independent signalling [56]. The CXCR4-β-arrestin complex is a potent inducer of the MAPK^p42/44 ^pathway via RAF-1 [59]. MAPK^p42/44 ^phosphorylation was observed in SDF-1α treated M13MDA435-1 hybrid cells. However, both G_αi _and G_αq _coupled GPCRs do also stimulate MAPK activation [54] and thus it remains unclear whether MAPK^p42/44 ^was activated in M13MDA435-1 hybrid cells in a heterotrimeric G-protein dependent or independent manner.

## Conclusions

In the present study we investigated the EGF- and SDF-1α mediated migratory behaviour of the two hybrid cell lines, derived from spontaneous fusion events [7,10], in relation to their parental cells. In accordance to a previous study [7] each hybrid cell line exhibited a unique migratory behaviour. Analysis of three major signal transduction cascades (PLC-γ1, PI3K/AKT, RAS-RAF-MAPK) indicated that the hybrid cells' unique migratory behaviour is likely attributed to a unique signal transduction cascade pattern in each hybrid cell line. Particularly of interest was the finding of the diametrically opposed L294002 effect on the migratory activity of M13MDA435-1 and -3 hybrid cells. Our data indicate that the Ly294002 mediated activation of the spontaneous as well as EGF and SDF-1α mediated migration of M13MDA435-1 hybrid cells was most likely attributed to an active RAF-AKT crosstalk in these cells. We thus conclude from our data that they nicely illustrate the random/unpredictable nature of cell fusion. Even though tumour hybrid cells may appear similar at a first glance, they might differ in fundamental properties, including differential kinetics of signal transduction cascades and cross-talks. Our data provide evidence that cell fusion between breast tumour cells and breast epithelial cells exhibiting stem cell properties can give rise to hybrid cells exhibiting a functional RAF-AKT crosstalk. Inhibition of the AKT dependent RAF block will restore RAF-1-MAPK^p42/44 ^signalling, which might result in a progression of these cells in case of an anti-AKT therapy. Thus, a thorough characterisation of tumour tissue samples, including overall expression of receptors and signal transduction molecules as well as their activation states, should be performed prior to tumour therapy.

## Methods

### Cell culture

The M13SV1-EGF-Neo breast epithelial cell line, exhibiting stem-like characteristics, [60] was kindly provided by James E. Trosko (Michigan State University, East Lansing, MI) and was cultivated in MSU-1 basal media (Sigma Aldrich, Taufkirchen, Germany) supplemented with 10% foetal calf serum (FCS) (PAA Laboratories, Linz, Austria), 1% Penicillin/Streptomycin (100 U/ml Penicillin, 0.1 mg/ml Streptomycin; PAA Laboratories, Linz, Austria), 10 μg/ml human recombinant EGF, 5 μg/ml human recombinant Insulin, 0.5 μg/ml Hydrocortisone, 4 μg/ml human Transferrin, 10 nM β-Oestrogen (all chemicals were purchased from Sigma Aldrich, Taufkirchen, Germany), and 400 μg/ml G418 (Biochrom AG, Berlin, Germany) at 37°C and 5% CO_2 _in a humidified atmosphere. The breast cancer cell line MDA-MB-435-Hyg was maintained in DMEM (PAA Laboratories, Linz, Austria) supplemented with 10% FCS,1% Penicillin/Streptomycin, and 200 μg/ml Hygromycin B (PAA Laboratories, Linz, Austria). M13MDA435-1 and M13MDA435-3 hybrid cells were cultivated in DMEM (PAA Laboratories, Linz, Austria) supplemented with 10% FCS,1% Penicillin/Streptomycin, 400 μg/ml G418 (Biochrom AG, Berlin, Germany), and 200 μg/ml Hygromycin B (PAA Laboratories, Linz, Austria) as described previously [7].

### Short tandem repeat analysis

STR analysis was performed as described previously [7]. In brief, genomic DNA of cells (1 × 10^6^) was isolated using the NucleoSpin^® ^Blood Kit (Macherey&Nagel, Düren, Germany) in accordance to the manufacturer instructions. Five polymorphic regions were amplified by PCR. For primer pairs and PCR conditions pleased refer to [7]. Separation of the PCR-fragments was performed using a four-color laser induced fluorescence capillary electrophoresis system (Prism 3130; ABI, Weiterstadt, Germany) utilizing GeneScan Standard ROX-500 for fragment length evaluation. Evaluation was done using Genemapper v2.03 evaluation software (ABI, Weiterstadt, Germany).

### Flow cytometry

Flow cytometry was performed on a FACScalibur flow cytometer (Becton Dickenson, Heidelberg, Germany). Cells (1 × 10^5^) were stained with the following antibodies: anti-EGFR (clone 528; Merck KGaA, Darmstadt, Germany), anti-c-erbB-2/HER2/*neu *(clone 9G6; Merck KGaA, Darmstadt, Germany), anti-erbB-3/HER3 (clone 298; Santa Cruz Biotechnology, Heidelberg, Germany) and PE-conjugated anti-CXCR4 (clone 44717; R&D Systems GmbH, Wiesbaden, Germany). Staining of intracellular CXCR4 was performed as described previously [36]. First, membrane bound CXCR4 was blocked with non-conjugated anti-CXCR4 (clone 44717; R&D Systems GmbH, Wiesbaden, Germany). Subsequently, cells were fixed with 4% paraformaldehyde and permeabilized with 0.5% Triton X-100. Cells were washed thoroughly with PBS and stained with PE-conjugated anti-CXCR4 (clone 44717; R&D Systems GmbH, Wiesbaden, Germany). Isotype matched antibodies served as control: IgG1 (Beckman Coulter, Krefeld, Germany) and PE-conjugated IgG2b (R&D Systems GmbH, Wiesbaden). In case of non-conjugated primary antibodies, cells were stained with a secondary PE-conjugated goat-anti-mouse antibody (R&D Systems GmbH, Wiesbaden, Germany). Data were analyzed using the WinMDI 2.8 software (Scripps Reserach Institute, La Jolla, CA, USA).

### Cell migration analysis

Cell migration analysis was performed by applying the 3D-collagen matrix migration assay combined with computer-assisted cell-tracking as described previously [7,36,42,61,62]. In brief, liquid collagen solution (Purecol; Nutacon BV, Leimuiden, The Netherlands) was mixed with 10 × MEM (Sigma Aldrich, Taufkirchen, Germany), 7.5% sodium bicarbonate solution (Sigma Aldrich, Taufkirchen, Germany) and cells (6 × 10^4^). Depending on the experimental setting EGF (100 ng/ml; Sigma Aldrich, Taufkirchen, Germany), SDF-1α (1 μg/ml; PAN Biotech, Aidenkirchen, Germany), Ly294002 (500 nM; VWR International GmbH, Darmstadt, Germany), and PD98059 (500 nM; VWR International GmbH, Darmstadt, Germany) were added to the solution. The collagen-cell suspension was filled in self-constructed cell migration chambers and the collagen was allowed to polymerize. Subsequently, cell migration chambers were put under a microscope into wooden boxes being tempered to 37°C. Cell migration within the 3D-collagen lattice was recorded for at least 15h by time-lapse video microscopy. For data analysis, 30 cells of each sample were randomly selected and two-dimensional projections of the paths were digitized in 15 min intervals.

### Calcium measurements

Changes in the intracellular calcium levels of cells were determined as described previously [36,47,61] in accordance to the method described by Gergeley et al. [63]. Cells (5 × 10^5^) were labelled with 4 μM Fluo-3 (Invitrogen). In dependence of the experimental setting, cells were stimulated with either 100 ng/ml EGF (Sigma Aldrich, Taufkirchen, Germany) or 0.1 μg/ml SDF-1α (PAN Biotech, Aidenkirchen, Germany). Substances were added after 50s. The tube was mixed, and acquisition was continued for a total of 204.80s. Measurements were performed using a FACScalibur flow cytometer (Becton Dickenson). For analysis, the mean fluorescence intensity (MFI) of 10s intervals was determined. The mean MFI of the first 50s of each calcium measurement without a stimulus was defined as a baseline level (mean MFI_baseline_) and was compared to mean MFI of the first 60s after stimulation (mean MFI_stimulation_), which was defined as the rate of calcium influx. The mean MFI_stimulation _of each calcium measurement was calculated in relation to the appropriate mean MFI_baseline _of the same experiment, which was set to 100%.

### Western Blot

To investigate the AKT and MAPK^p42/44 ^phosphorylation levels in dependence of EGF and SDF stimulation as well as in the presence of the PI3K inhibitor Ly294002 and the MAPK^p42/44 ^inhibitor PD98059 1 × 10^5 ^cells were treated with 100 ng/ml EGF (Sigma Aldrich, Taufkirchen, Germany) or 1 μg/ml SDF-1α PAN Biotech, Aidenkirchen, Germany) for 5 minutes or were pretreated with 500 nM Ly294002 (VWR International GmbH, Darmstadt, Germany) or 500 nM PD98059 (VWR International GmbH, Darmstadt, Germany) for 30 minutes. Samples were separated by SDS-PAGE on a 10% SDS polyacryamide gel and transferred to PVDF nitrocellulose membranes (Millipore) under semi-dry conditions. Membranes were blocked overnight with 10% (w/v) non-fat dry milk in TBS-T. MAPK^p42/44^, pMAPK^p42/44^, AKT, pAKT S473, RAF-1, pRAF-1 S259, and elf4E were detected by using the following antibodies: MAPK^p42/44 ^(rabbit polyclonal; Cell Signalling, New England Biolabs, Frankfurt am Main, Germany), pMAPK^p42/44 ^(rabbit polyclonal; Cell Signalling, New England Biolabs, Frankfurt am Main, Germany), AKT (clone 11E7; rabbit monoclonal, Cell Signalling, New England Biolabs, Frankfurt am Main, Germany), pAKT S473 (clone D9E; rabbit monoclonal, Cell Signalling; New England Biolabs, Frankfurt am Main, Germany), RAF-1 (rabbit polyclonal, Cell Signalling; New England Biolabs, Frankfurt am Main, Germany), pRAF-1 S259 (rabbit polyclonal, Cell Signalling; New England Biolabs, Frankfurt am Main, Germany), and elf4E (rabbit polyclonal; Cell Signalling, New England Biolabs, Frankfurt am Main, Germany). For detection of primary antibodies the HRP-conjugated secondary anti-rabbit IgG (Cell Signalling, New England Biolabs, Frankfurt am Main, Germany) was used. Bands were visualised using the LumiGLO^® ^Reagent (Cell Signalling, New England Biolabs, Frankfurt am Main, Germany) or the BM Chemiluminescence Western Blotting Substrate (POD) (Roche Diagnostics Geutschland GmbH, Mannheim, Germany) in accordance to the manufacturers' instructions. Blots were detected with the Aequoria Macroscopic Imaging system (Hamamatsu Photonics Germany, Herrsching am Ammersee, Germany) or by conventional exposure to Kodak T-MAT Plus DG X-ray films (Röntgenversand Wurzbacher, Obernissa, Germany).

## Competing interests

The authors declare that they have no competing interests.

## Authors' contributions

CÖ performed the experiments. JS characterised the hybrid cell lines. SMS and BHB: performed and analysed the STR data analysis. BN analysed the migration data. KSZ wrote and corrected the manuscript. TD designed the experiments, wrote and corrected the manuscript. All authors have read and approved the final manuscript.
